# A 13 year hospital based study on the Trend of Urinary Stone Disease in Uttarakhand, India

**DOI:** 10.3126/nje.v11i1.35896

**Published:** 2021-03-31

**Authors:** Monica Kakkar, Rakesh Kakkar

**Affiliations:** 1 Department of Biochemistry, NRI Medical College, Guntur, Andhra Pradesh, India; 2 Department of Community and Family Medicine, All India Institute of Medical Sciences, Mangalagiri, Guntur, Andhra Pradesh, India

**Keywords:** Renal stones, Urinary calculus, Urinary Stone disease, Urolithiasis

## Abstract

**Background:**

The present retrospective study on urinary stone disease in the Uttarakhand state was necessitated as no study has been done yet.

**Methods:**

A 13 year retrospective study (from 2005 to 2018) was conducted on the urinary stones removed from the patients, admitted at Himalayan Institute of Medical Sciences, Dehradun. The incidence of the disease, site of stones in urinary tract upon diagnosis, composition of removed stones and occurrence of a possible co-relationship between the incidence of the urinary stone disease at different times, age, sex, religion of the patients was investigated.

**Results:**

The frequency of occurrence of urinary stones in males was found to be almost three times more as compared to their female counterparts. The above trend was consistent over the entire period of the study. Interestingly, in the Muslim and Sikh population of the area, females were found to be less prone to the problem as compared to their Hindu counterparts. However, in all religious groups, 21-40 years old subjects were found to be most susceptible to the problem and approximately 90% of the urinary stones were recovered from the kidneys and primarily composed of calcium oxalate.

**Conclusion:**

The co-relationship between the occurrence of urinary stones with age, sex of the patients, their religion & site of stones on diagnosis was found to be statistically significant.

## Introduction

During the last 30 years, a significant increase in the frequency of occurrence of urinary stone disease in the world’s population has been reported [1].The increase in this trend of urinary stones disease has been attributed to the changes in the life styles of the people and the environment, in addition to the indiscriminate use of medicines by self-medication [2]. Dietary habits and the fluid intake have been thought to be main etiological causes influencing the formation of stones in the urinary tract of human beings. The amount of fluid intake influences calculi formaton by changing the concentration of the stone forming constituents in the urine [1]. Men are 3 times more susceptible to the disease as compared to the females of the identical age group [3]. Age, sex, geographical location, family history and nutritional status of the population have been thought to be primarily responsible for the formation of urinary stones. As the urinary stone disease is multifactorial in nature, hence, it is practically impossible to pinpoint a definite etiological factor. The problem gets further complicated by its high frequency of recurrence, which is reported to be 35, 74 and 98 percent between 1-3, 1-10 and 1-25 years respectively [4].The frequency of occurrence of urolithiasis has been shown to vary from country to country, region to region, between races, sexes and the age of the patients[5]. Approximately five to twelve percent of the global population is known to develop this disease during their life time. In 2000, approximately 40 percent of the population of the world in the high-risk zones (Asian countries especially Saudi Arabia and India) has been reported to be affected by this disease and this percentage is expected to grow up to 50 percent by 2050 [6]. Nearly, one percent of all hospital admissions have been reported to be due to urinary stones and 10 percent of the renal stone disease cases end up into renal failure [7]. In the various regions of the world, the percentage of population reported to be affected by urinary stone disease is: Asia (2-5%), Europe and North America (8-15%) and South Arabia (20%) [8].

In Asia the stone belt countries are India, Pakistan and Southern China. Approximately 15 percent of the Indian population has been reported to be affected by this disease. The occurrence of this problem was found to be highest in the Northern, Western and Central regions of the country. The incidence of this disorder is moderate in Deccan Plateau and very low in Southern coastal parts of the country [9]. Based upon the incidence of the occurrence of the problem, India is divided into two Stone Belt areas. North India forms the first stone belt. This belt starts from Jammu & Kashmir and passes through Punjab, Haryana, Delhi and ends up in Uttar Pradesh. Second stone belt starting from Gujarat terminates in Jabalpur (Madhya Pradesh) [10].

The northern state of Uttaranchal having its capital at Dehradun was carved out from the state of Uttar Pradesh on 9th November 2000. Its name was changed to Uttarakhand in January 2007. The retrospective, epidemiological studies in the state of Uttarakhand assumes significance as a sea change has taken place in the living conditions, working habits and the nutritional status of the population over last 20 years. The present study was conducted, as no significant study has yet been reported in literature on the urinary stone disease prevalence in the state of Uttarakhand.

## Methodology

### Study design and participants

The present investigation is a retrospective observational study undertaken in Biochemistry Department of Himalayan Institute of Medical Sciences, Dehradun.

### Data Collection

Urinary stone analysis was started in the Clinical Biochemistry laboratory of the Biochemistry Department in November 2004. Data for the study on stone analysis was retrospectively collected from the previously available records from January 2005 to December 2008. Moreover data for the period from 2009 to 2018 is based upon the urinary stone analysis conducted by the first author. To collect data for the age, sex, religion, complete history of the patients, sites of stones upon diagnosis, chemical composition of the recovered stones and the various investigative procedures /surgery, a predesigned format was used. For chemical analysis the urinary stones were cut, crushed and converted into the powdered form. To qualitatively determine the composition of the urinary stones with respect to Calcium, Phosphate, Oxalate, Uric acid, Cystine and Ammonium salts, Hodgkinson procedure (using analytical reagent grade chemicals) was followed [11].

### Inclusion and exclusion criteria

During the period of approximately thirteen years (January 2005 to December 2018), all the urinary stone disease patients, who visited the Urology OPD of Himalayan Institute of Medical Sciences and their urinary stone samples were received in the Clinical Biochemistry lab for chemical analysis, were included in the present study. The patients whose chemical analysis data of the recovered stone was either missing or incomplete were excluded from the study.

### Ethical Clearance

The study was started after obtaining approval from the Ethical Committee of Himalayan Institute of Medical Sciences vide their letter no: SRHU/HIMS/RC/2017/165, PIN- Biochemistry/2017/02.

### Sample size calculation

Epidemiological studies have demonstrated that the prevalence of the renal stone disease is approximately 15% in Northern India [12]. Applying the n=z^2^*P (1-P)/d^2^ formula, with the confidence interval and precision of 95 and 5 percent respectively, the required minimum sample size was found to be 196. However, in the present study all the 435 kidney stone patients who were registered in the Urology OPD of Himalayan Institute of Medical Sciences and having the requisite records available, were included.

### Data management and statistical analysis

Descriptive analyses were reported as frequencies and percentages for categorical variables. Variables of interest were compared and analyzed with respect to gender i.e. male vs. females and age groups i.e. <20, 21-40, 41-60 and >60 years. Chi-square test was applied to analyse the differences in categorical variables between respective groups. For the expected cell frequencies below 5, categorical variables were subjected to Yates’ corrected chi-square. Two-tailed P value of <0.05 was taken to be statistically significant. Statistical Package for the Social Sciences (SPSS Version 20.0; IBM Chicago, USA), was used to analyze the data.

## Results

### Demographic characteristics Religion and Gender

**Figure 1:**
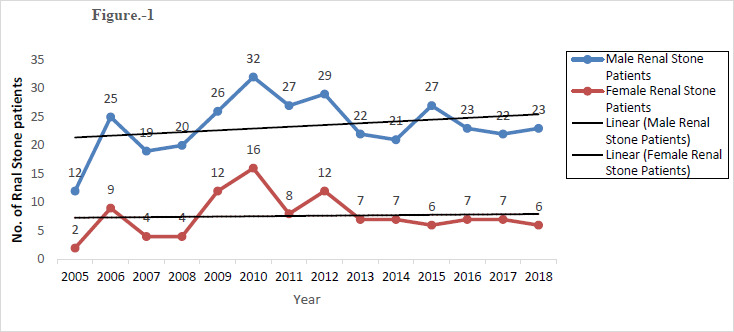
Yearly record of Renal Stone patients admitted HIMS, Dehradun

Results presented in Table 1 and Fig. 1 record the data on 435 urinary stone patients, who visited the Urology OPD of Himalayan Institute of Medical Sciences with urinary stone disease over a period of approximately thirteen years (January 2005 to December 2018). Out of 435 urinary stones, 328 were recovered from the males and 107 from the female patients. From Table-2 it can be concluded that a highly significant (p-value < 0.05) positive co-relation exists between the occurrence of stone and time and also between the presence of renal stone disease between the sexes.

Table-3 shows that a significant association exists between the occurrence of urinary stones and the religions of the patients according to gender. Higher proportion of Hindu females (86.9% vs. 68.6%; p=0.001) had urinary stones as compared to males. On the other hand, Muslim, Sikh and Christian males were more likely to have urinary stones than females (p=0.001). The Male/ Female ratio of urinary stones in Hindus, Muslims, and Sikhs patients was found to be 2.4:1, 4.5:1 and 17.3:1 respectively.

### Location of renal stones in the urinary tract

Table-4 shows out of 328 urinary stones obtained from the male patients, 273, 38, 3, 5, 4 and 5 stones were recovered from the kidneys, ureters, urethra, bladder, uretero-vesical junction (UVJ) and pelvi-ureteric junction (PUJ) respectively. In the case of 107 urinary stones obtained from the female patients, 100, 5 and 2 stones were recovered from the kidneys, ureters and bladder respectively. The two groups were comparable with respect to the site of stones recovered from the urinary tract (p=0.40). In case of female subjects out of 107 stones, no stone was recovered from either the urethra, UVJ or PUJ. Interestingly out of a total of 273 kidney stones in males and 100 kidney stones in females only 3 stones in both the sexes were found to be Staghorn in nature. The bilateral, right side (kidney, ureter, UVJ and PUJ), left kidney (kidney, ureter, UVJ and PUJ), bladder and urethral origin of the urinary stones was found to be in 15.0, 35.3, 44.5, 2.9 and 0.6 percent patients respectively. Only 1.7 percent of urinary stones were found to be recurrent in nature. The Male/ Female ratio was found to be 2.7:1, 7.6:1 and 2.5:1 in renal, ureteric and bladder stones respectively.

### Age

Studies presented in Table-5 revealed that out of 328 male patients, 32, 169, 104 and 23 male subjects were found to have urinary stones at the age less than 20, between 21-40, between 41-60 and more than 60 years respectively. Out of 107 female patients, 12, 44, 42 and 9 female subjects were found to have urinary stones at the age less than 20, between 21-40, between 41-60 and more than 60 years respectively. Table-5 also records the relationship between age, sex and religion of patients with occurrence of urinary stones in both sexes of Hindu, Muslim, Sikh and Christian population in Uttarakhand state. The highest male / female sex ratio (3.8:1) for the incidence of urinary stones was also found in the people of 21-40 years age group.

### Chemical nature of renal stones

Results (Table-6), demonstrate that more than 90 percent of the stones recovered both from the male and the female patients were found to be calcium oxalate in nature. The chemical nature of remaining 10 percent of stones in both the sexes was either found to be Calcium Phosphate, Struvite, Urate or Mixture of various salts.

## Discussion

Urinary stone disease, not only directly affects the health of the patients but it also indirectly affects the economy of the country. As the highest frequency of occurrence and recurrence of this disease is present in people when they are in their most productive age group of 21-40 years, hence, the effective management of the disease assumes significance particularly in areas which fall in the high incidence stone belt region of the country [13,14]. Complete management of the disease includes proper evaluation,the prophylactic measures taken to prevent its occurrence or recurrence and its treatment. For the effective management, it is important to conduct an epidemological study, not only on the composition and the site of stone upon diagnosis but also to compare its frequency of occurrence in diffferent ethenic groups so as to establish if, any possible relationship exists between the prevalence of the disease and the diverse dietary habbits of various religious groups.Until 1980, treatment of urinary stones primarily involved open surgery. During the last 30 years or so, revolution has has taken place in the management of the disease of urinary stones without involving open surgery. Various techniques like, percutaneous nephrolithotomy (PCNL), extracorporeal shock-wave lithotripsy (ESWL) and ureterorenoscopy (URS) have been developed which use various types of energies to fragment the stones [15].

### Demographic characteristics Religion

Thirteen years (January 2005 to December 2018) retrospective, epidemiological study was conducted on435 urinary stone disease patients of Uttarakhand region who visited the Urology OPD at Himalayan Institute of Medical Sciences, Dehradun. Out of these 435 patients, highest percentage of urinary stones patients were found in Hindus (73.10%) followed by Muslims (14.0%), Sikhs (12.6%) and Christians (0.3%). Interestingly, the frequency of occurrence of urinary stones in various religious groups, observed during the present study, was found to have an identical trend with the percentage population of various religious groups (Hindus-82.6%, Muslims-13.9%, Sikhs-2.67% and Christians-0.37%) as per the 2011 census of Uttarakhand [16].

### Gender of patients

The present study further revealed that in all the religions, males were found to be 3 times more prone to the formation of urinary stones as compared to their female counterparts (Tables 1-2 &Fig-1).The above observation is in complete agreement with the conclusion drawn on the prevalence of the disease in the sexes based upon the concentration of biomolecules, present in the urine of both the sexes, which act as potent inhibitors of in vitro inhibitors mineralization. These studies [17,18] demonstrated that the level of urinary inhibitor of in vitro mineralization is three times higher in female renal stones patients as compared to their male counter parts of the identical age group. In both the sexes trend of urinary stones seems to increase with time (Fig-1).Interestingly with respect to the percentage of occurrence of stones within sexes, as compared to the males (73.1%) more females (82.97%) were found to have urinary stones in Hindus and least in the case of Sikhs where 12.6 percent males and 2.64 percent females were found to have urinary stones.

### Age

In both sexes not only the highest occurrence of the disease but also the highest male/ female sex ratio with respect to the disease was found in the population between the ages of 21-40 years (Table-5). However, in Muslims, the highest male/ female ratio occurred in the population having age between 41-60 years (Table-5). The above observation gets supported from the study on urinary inhibitors, which demonstrated that differences in the concentration of inhibitors of mineralization present in the urine samples of male and females was found to be minimum in children and patients of more than 60 years of age as compared to the patients having age between 20-40 years [18]. These differences, in the level of urinary inhibitors, were postulated to be due to the difference in the level of sex hormones present in different age groups. There seems to be no plausible explanation available to explain the observed differences prevailing in various religions, however, religious prejudices coupled to religion based under reporting tendencies of various age groups could possibly be responsible for the above observed differences.

### Urinary tract location of stones

Table-4 demonstrates that stones can be formed in all parts of the urinary tract, however, their highest percentage in males (83%) and females (93%) was found in the kidneys. Interestingly highest male / female ratio was found in the case of ureteric stones as compared to stones occurring in different locations in the urinary tract where this ratio favorably compared with the overall ratio of 3:1 between the males and females. Highest percentage (44.5%) of urinary stones were recovered from the left side (kidney, ureter, UVJ and PUJ) followed by those recovered from the right side (kidney, ureter, UVJ and PUJ) (35.3%), bladder (2.9%) and urethral (0.6%). The percentage of bilateral and recurrent stones was found to be 15 percent and 1.7 percent respectively. Review of literature revealed that neither any such observation has been reported previously nor any explanation is readily available to explain the above results.

### Prevalence of Staghorn stones

Out of 328 male and 107 female urinary stone patients, only in three patients of both the sexes, the nature of the stones was found to be Staghorn (Table-6).Studies by Rieu have shown that with early and effective management the percentage occurrence of Staghorn stones has decreased from 20 to <4%[19]. Improvement in management practices coupled with underreporting could perhaps be responsible for the observed lower prevalence of Staghorn stones.

### Chemical analysis of urinary stones

Analysis of the stones composition revealed that approximately 90 percent of the urinary stones were found to be Calcium Oxalate (CaOx) in nature. Out of these 90 percent CaOx stones, approximately 30, 10 and 50 percent stones were found to consist of Calcium Oxalate Monohydrate, Calcium Oxalate Dihydrate and a mixture of the two Calcium Oxalate forms respectively. Ten percent of remaining urinary stones were found to either consist of Calcium Phosphate, Urate, Struvite or a mixture of various above salts. This is consistent with the results obtained on the composition of the stones recovered from the urinary tract by various other workers which also demonstrated the presence of the same trend of chemical composition occurring in urinary stones isolated from the patients from various regions of the world [20,21, 22, 23]. Knowing the chemical composition of the stones recovered from the patients of a specific area and relating to it with their frequency of occurrence can help the clinicians for designing specific management strategies. Urinary stones were found to be of various colours. The colour of the highest percentage of stones was found to be brownish (59%) followed by whitish (20%), blackish (9%), greyish (6%), yellowish (4%), orange (1%) and colourless (1%). Studies [24]also showed that the colour of the urinary stones can either be dark brown, dirty white, yellow, red, pink, or black and the nature of the colour is influenced by the constituents of stones and the occurrence of infection.

More such data from other tertiary care centers of Uttarakhand state and also from other states falling in the stone belt regions of India will be required before conclusively concluding that the prevalence of this disease can either be attributed to ethnicity or nutritional habits of the population. The results obtained from such comprehensive studies can go a long way in helping the urologist of the region to design various strategies for managing the problem of urinary stones in the population of their respected areas.

## Limitation of the study

After the creation of the Uttarakhand state a lot of development and improvement in the socioeconomic status of the people has taken place. In addition to the above facts a significant increase in the medical facilities also took place in the form of creation of more hospitals and super specialties. Keeping the above factors in view, in order to obtain a real idea about the epidemiological status of urinary stone disease in the state, it is highly desirable that a parallel study be conducted covering all the important Hospitals and super-specialties available in the state. The data, thus collected would help us to prepare a state registry of urinary stone disease, which could act as a guide line for designing suitable effective management practices.

Religious prejudices coupled to religion based under reporting tendencies of various age groups could also have an impact on the observations made during the present study.

## Conclusion

A statistically significant co-relationship was observed between the prevalence of urinary stones with age, sex of the patients, their religion & site of the urinary stones on diagnosis. About 90 percent of the urinary stones were recovered from the kidneys and consisted of Calcium Oxalate.

### Future scope of the study

Studies covering the following points may help physicians to plan suitable management strategies for urinary stone disease especially in the stone belt regions of the country.

- Since nucleation and crystal growth are controlled by different kinetic and bio-regulatory mechanisms, it will be of interest to determine and compare the chemical composition of the small stones (mostly <5 mm in male and <8mm in females) with the bigger stones recovered after surgical interventions.
- Investigating the layer-wise constituents of the stones would provide information on the mechanisms of initial nucleation and subsequent growth involved in stone formation.
- Conducting identical studies in other states on the incidence and composition of urinary stones and their possible relationship with sex, age, religion and nutritional habits of their population.

### What is already known on this topic?

1. The incidence of the disease is approximately three times more in males as compared to the female of the same age group.
2. Most of the urinary stones are Calcium Oxalate in nature

### What this study adds

Review of literature revealed the following:

1. As no such comprehensive study covering a long duration has ever been conducted in the state of Uttarakhand, hence, the present study is first of its kind to establish a possible between occurrence of urinary stone disease with age, sex, site in urinary tract and chemical composition of the urinary stones in the state.
2. As no reference is available in literature which compares the frequency of occurrence of urinary stones in different religions with respect to differences in sexes and age groups, hence, its uniqueness.

## Figures and Tables

**Table 1:** Yearly record of Urinary Stones patients admitted at Himalayan Institute of Medical Sciences, Dehradun

Year	Total Patients (n=435, %)	Male (n=328, %)	Female (n=107, %)
**2005**	14 (3.2)	12 (3.6)	2 (1.8)
**2006**	34 (7.8)	25 (7.6)	9 (8.4)
**2007**	23 (5.3)	19 (5.8)	4 (3.7)
**2008**	24 (5.5)	20 (6.1)	4 (3.7)
**2009**	38 (8.7)	26 (7.9)	12 (11.2)
**2010**	48 (11.0)	32 (9.7)	16 (14.9)
**2011**	35 (8.0)	27 (8.2)	8 (7.5)
**2012**	41 (9.4)	29 (8.8)	12 (11.2)
**2013**	29 (6.6)	22 (6.7)	7 (6.5)
**2014**	28 (6.4)	21 (6.4)	7 (6.5)
**2015**	33 (7.6)	27 (8.2)	6 (5.6)
**2016**	30 (6.9)	23 (7.0)	7 (6.5)
**2017**	29 (6.6)	22 (6.7)	7 (6.5)
**2018**	29 (6.6)	23 (7.0)	6 (5.6)

**Table 2:** Statistical Analysis: Chi-Square Tests on year-wise cases of both sexes

	Value		Df		Asymp. Sig. (2-sided)	
	Males	Female	Males	Females	Males	Females
**Pearson Chi-Square**	22.516	22.516	12	12	**0.032**	**0.032**
**Likelihood Ratio**	23.662	23.662	12	12	0.023	0.023
**Linear-by-Linear Association**	1.666	1.666	1	1	0.197	0.197
**N of Valid Cases**	107	107				

**Table-3:** Association between the Urinary Stone Disease and the religion of the Patients

S.No.	Patients admitted	Male	Female	Percentage of Total		P value
		328	107	Stone Patients	2011 State Population Census	
**1.**	Hindus	225 (68.6%)	93 (86.9%)	73.1	82.97	0.001
**2.**	Muslims	50 (15.2%)	11 (10.2%)	14.0	13.97	0.001
**3.**	Sikhs	52 (15.9%)	3 (2.8%)	12.6	2.64	0.001
**4.**	Christians	1 (0.3%)	0 (0.0%)	0.3	0.37	0.001

**Table-4:** Site of stones recovered from the Urinary Tract

S No	Physical Location	Sex of Patients		Sex Ratio (Male/Female)	P-value
		Male	Female		
**1**	Kidneys*	273	100	2.7:1	0.40
**2**	Ureter	38	5	7.6:1	0.40
**3.**	Urethra	3	0		
**4**	Bladder	5	2	2.5:1	0.40
**5**	UVJ	4	0	-	
**6**	PUJ	5	0	-	

* Six Kidney Stones (3 in Male and 3 in Female) were of Stag horn Type.

**Table-5:** Relationship between Occurrence of Urinary Stones with Age, Sex & Religion of Patients

S.No	Total Patients in each Sex/ sex ratio	Patients Age (years)			
		<20	21-40	41-60	>60
**1**	Total Males patients (n=328)	32	169	104	23
	a) Hindus	25	119	64	17
	b) Muslims	6	23	19	2
	c) Sikhs	1	27	20	4
	d) Sikhs	0	0	1	0
**2.**	Total Females patients (n=107)	12	44	42	9
	a) Hindus	10	35	40	8
	b) Muslims	1	8	2	0
	c) Sikhs	1	1	0	1
	d) Sikhs	0	0	0	0
**3.**	Males/Female ratio	2.7:1	3.8:1	2.5:1	2.6:1
	a) Hindus	2.5:1	3.4:1	1.6:1	2.1:1
	b) Muslims	6.0:1	2.9:1	9.5:1	-
	c) Sikhs	1:1	27:1	-	4.0:1
	d) Sikhs	-	-	-	-

**Table-6:** Chemical Composition of the recovered Stones

S No	Stone Type	Number of Patients	
		Male (% of Total)	Female (% of Total)
**1**	Calcium Oxalate	221 (90.1)	66 (91.7)
	a) Mainly CaOx Monohydrate	(28.3)	(30.6)
	b) Mainly CaOxDihydrate	(7.0)	7 (9.7 )
	c) Mixture of CaOx Mono & Dihydrate	135 (55.3)	37 (51.4)
**2**	Calcium Phosphate (Apatite & Hydroxy apatite)	6 (2.5 )	2 (2.8 )
**3**	Struvite	5 (2.0 )	1 (1.4 )
**4**	Uric Acid	4 (1.6)	1 (1.4 )
**5**	Mixed	8 (3.3)	2 (2.8 )
**6**	Stone amount not sufficient for analysis	5 (2.0)	1 (1.4)
